# Change in substance use among patients in opioid maintenance treatment: baseline to 1-year follow-up

**DOI:** 10.1186/s12954-024-01005-x

**Published:** 2024-05-24

**Authors:** Endre Dahlen Bjørnestad, John-Kåre Vederhus, Thomas Clausen

**Affiliations:** 1 Addiction Unit, Sørlandet Hospital HF, Po. box 416, Kristiansand, Norway; 2 Norwegian Centre for Addiction Research (SERAF), University of Oslo, Kirkeveien 166, Oslo, N-0407 Norway

**Keywords:** Substance use disorder, Opioid use disorder, Cognitive impairment, Opioid maintenance treatment

## Abstract

**Background:**

Individuals with opioid use disorder (OUD) often have concurrent use of non-opioid substances. When patients enter opioid maintenance treatment (OMT), less is known about outcomes regarding the use of other types of drugs. Here we aimed to investigate changes in substance use among patients entering outpatient OMT, from treatment initiation to 1-year follow-up.

**Methods:**

We used data from the prospective Norwegian Cohort of Patient in OMT and Other Drug Treatment Study (NorComt). Among 283 patients who entered OMT at participating facilities across Norway, 179 were assessed at follow-up. Of these patients, 131 were in a non-controlled environment, and were included in the present analysis. The main outcome was change in substance use. Logistic regression analysis was applied to identify factors associated with abstinence from all substances (other than agonist medication) at follow-up.

**Results:**

Along with opioid use, most patients reported polysubstance use prior to entering treatment. No significant differences were found in baseline characteristics between the included and non-included groups when examining attrition. At the 1-year follow-up, reduced substance use was reported. While in treatment, around two-thirds of patients continued using other drugs to varying degrees. At follow-up, about one-third of patients reported abstinence from all drugs, apart from the agonist medication. Factors related to abstinence included a goal of abstinence at baseline (OR = 5.26; 95% CI 1.14–19.55; *p* = 0.013) and increasing age (OR = 1.05; 95% CI 1.00–1.09; *p* = 0.034).

**Conclusions:**

The majority of patients entering OMT used other substances in addition to opioids. About one-third of patients reported abstinence at the 1-year follow up. Although the majority of patients continued co-use of other drugs while in treatment, for most substances, less than 10% reported daily use at follow-up, with the exception of cannabis which was used daily/almost daily by about 2 in 10. Higher age and treatment goal at the start of OMT were important factors related to reducing concomitant substance use during treatment. These findings suggest that many patients entering OMT are in need of treatment and support related to the use of other substances, to further improve prognosis.

**Clinical trial registration:**

Clinicaltrials.gov no. NCT05182918. Registered 10/01/2022 (the study was retrospectively registered).

**Supplementary Information:**

The online version contains supplementary material available at 10.1186/s12954-024-01005-x.

## Background

Opioid maintenance treatment (OMT) is an evidence-based treatment model aimed at reducing harmful effects associated with non-medical opioid use for individuals affected by opioid use disorder (OUD) [1–3]. Several studies have demonstrated that OMT has a positive influence on a range of outcomes, including reducing non-medical opioid use [4], overdose risk [5], mortality [6, 7], morbidity [8], and crime [9], as well as improving quality of life [1]. However, less is known about how OMT affects the use of other substances [10]. Individuals in OMT often have additional substance use disorders (SUDs) alongside their OUD [11, 12]. Although opioid agonist medication may help individuals stabilize their OUD, it will not necessarily affect other SUDs. Polysubstance use is quite common among persons with OUD [12–14], and is generally associated with a range of negative health outcomes [15], and with poorer prognosis in OMT [14–18]. Aging OMT populations have been described in several countries, adding new challenges for patients and treatment providers [19–22]. The wide range of morbidities associated with OUD imposes a heavy burden on patients along their path towards recovery [1, 13, 23].

Individuals with SUD are also commonly burdened with high levels of mental distress, which can negatively impact treatment outcomes [13, 23]. Benzodiazepine use among patients receiving opioid agonist treatment is a topic of much debate [24–26]. On one hand, benzodiazepines may provide relief from a range of anxiety-related psychiatric symptoms that are common among patients in opioid agonist treatment [26]. On the other hand, benzodiazepines can have sedative effects, interact with other substances, and increase the risk of overdose and other negative outcomes [26, 27]—although evidence of association with increased mortality remains inconclusive [28]. OMT is generally provided in outpatient settings, although some patients also enter inpatient treatment at the time of starting agonist treatment, or during the treatment trajectory. Most patients enter OMT with the aim of alleviating OUD-associated problems, but their specific treatment goals vary. Some aim for full rehabilitation, including abstinence from non-medical use of opioids and other drugs, while others may aim to stabilize their current non-medical opioid use, and other drug use, without necessarily having an ambition of abstinence. Both treatment goals are appropriate, including that of harm reduction, but they diverge in terms of the degree of rehabilitation effort provided and in the outcomes for patients [29]. Being abstinent from all non-medical drug use can be considered a preferred outcome of OMT [30–32]. The co-use of other substances may impair treatment outcomes [4], such that it is important to explore factors related to abstinence, i.e., use of only agonist medication.

In the present study, we aimed to investigate changes in substance use among patients entering OMT programs, with comparison between treatment initiation versus 1-year follow-up. To this end, we described substance use among patients entering outpatient OMT (T0), investigated changes in the use of substances reported at the start of treatment (T0) to the 1-year follow-up (T1), and described the substance use patterns at the 1-year follow-up (T1). Additionally, we explored factors associated with abstinence from substance use (apart from OMT medications) at T1.

## Methods

### Study design

We used data from the Norwegian Cohort of Patients in OMT and Other Drug Treatment (NorComt) study [9, 33]. NorComt is a longitudinal, naturalistic, multi-site study designed to increase our understanding of factors influencing treatment adherence and outcomes, within a diverse patient population, across the range of standard care treatment modalities. The baseline data collection period was from December 2012 to March 2015, with follow-up extending into 2016. In our present study of changes in substance use in an outpatient setting, our primary patient group of interest included individuals who entered OMT and were followed-up after 1-year.

### Setting

Participants were recruited from participating OMT facilities across Norway. In the Norwegian setting, OMT is mainly provided on an outpatient basis by publicly funded health services, following national treatment guidelines [34]. The OMT guidelines in use during the time period of the present study were implemented in 2010. The only criterion for entering treatment is an established OUD diagnosis. The study setting has previously been described in detail [9, 35, 36]. OMT is provided in collaboration with the primary healthcare and social services, with the specialist healthcare service as the overall responsible provider.

### Participants

The only formal inclusion criterion for participation in the study was admittance to an OMT treatment facility. There were no formal exclusion criteria. Participants were consecutively enrolled in the study when beginning treatment (T0). At baseline, they consented to be contacted one year later for additional data collection through a follow-up interview (T1) [29]. At each treatment center, clinicians conducted the baseline interviews of consecutively enrolled patients— a median of 18 days from treatment initiation. Follow-up data (T1) were collected at 12 months (range 11–18 months) following inclusion. In the present study, we focused on substance use in the 4 weeks prior to T0 and T1.

We aimed to include patients who started OMT and were available for the follow-up interview at T1. Patients who were assessed at T1 were excluded from analysis if they had been in a controlled environment (e.g., prison, inpatient SUD treatment, inpatient psychiatric treatment, etc.) within the 30 days prior to T1, unless it was only for a short detoxification period. The rationale was to include only patients who had no specific restrictions on substance use.

### Measures

The structured interview included questions about sociodemographic variables, housing, substance use, and a variety of measurements related to the treatment of substance use disorders, including prior treatment enrollment [31, 32]. Changes in substance use were our main outcome. The time periods for evaluation were the 4 weeks prior to T0 and T1. Patients were first asked to report their most used substances or addictive prescribed medications for a longer time-frame (i.e., during the 6 months prior to T0 or T1), and were then asked how much they had used these substances within the past 4 weeks. The form was limited to the four most used substances. Substances reported at T0 and not reported at T1 were coded as “No use”. Substance use was scored using a 6-point response format, ranging from 0 (indicating “No use”) to 5 (indicating “Daily use”). Patients were also asked the number of different substances they had used during the past 6 months. Polysubstance use was defined as reporting the use of two or more substances. To facilitate interpretation, we examined changes in main substance categories, with some related substances combined into a main category. The opioid use category included heroin and other opioids obtained without prescription (“unprescribed”). Amphetamines, cocaine, crack, methylphenidate, and other stimulants were combined into the “stimulant” category. Benzodiazepines were classified as either prescribed or unprescribed, and patients who reported using both categories were assigned to the unprescribed category. Prescribed benzodiazepines were separately analyzed because the OMT guidelines generally recommended against benzodiazepine use, even when prescribed. Therefore, we wanted to investigate whether there was a change in both benzodiazepine categories separately at follow-up. We also included intravenous use in the past 6 months.

Abstinence was defined as use of only the agonist medication. To analyze associations between covariates and abstinence, we selected the patients who reported “no use in past 4 weeks” at T1, and generated a dichotomized outcome variable consisting of “abstinence: yes or no”.

As a severity measure, we used the Severity of Dependence Scale (SDS) [37], which is a validated five-item scale designed to measure dependence on specific substances the past 4 weeks (e.g., “Did you think your use of amphetamines was out of control?”). In the present study, we used a version that was rephrased to reflect general dependence on substances the past 4 weeks (e.g., “Did you think your use of substances was out of control?”). Patients responded using a 4-point format, ranging from 0 (indicating “Never”) to 3 (indicating “Always”). The summed scale ranged from 0 to 15, with higher scores representing higher severity.

As a measure of mental distress past week, we used the Hopkins Symptom Checklist 25-item version (HSCL-25), with responses given using a 5-point Likert-type format [38, 39]. With this version, a score of 1.0 indicates mental distress of clinical concern [40]. Patients were also asked whether they have had a stable housing situation during the past 4 weeks, which was answered using a “yes-no” response format.

Patients’ treatment goals reported at baseline were used as an indicator of the patient’s overall ambitions at the start of treatment. Patients did not have to state a specific abstinence goal to receive treatment. Patients were asked “What is your goal with this treatment?” and the possible responses were “Rehabilitation with abstinence” (reflecting an ambition to use only the agonist medication over time) or “Stabilization and better control of substance use” (reflecting an ambition to merely reduce harms associated with substance use) [34].

Research has indicated that type of social network is associated with substance use [41]. The patients’ primary social networks were evaluated using a question from EuropASI [42]. Participants were asked with whom they had spent most of their free time the past 6 months, and the response options included family (with or without problem use of alcohol, medications, and substances), friends (with or without problem use of alcohol, medications, and substances), or being mostly alone. We recoded these responses such that participants were placed into one of three categories: having a primarily substance-using social network (family or friends with problem substance use), having family or friends without problem substance use, or being mostly alone.

### Statistical analysis

Participant sociodemographic data, substance-use variables, and health-related variables are presented descriptively. Continuous variables are reported as mean (M) ± standard deviation, or median (Mdn) and interquartile range (IQR). Categorical variables are reported as frequencies and percentages. Changes in ordinal outcomes were tested using the matched-pairs Wilcoxon signed-rank test. The associations between abstinence and relevant independent variables were investigated using logistic regression. Due to the limited sample size and the recommendation to have at least 10 observations per estimated parameter in each group in a multivariable logistic regression model [43], we first investigated relevant variables using bivariate analysis. Variables with a p value of < 0.2 were included in further analysis [44].The final multivariable logistic regression was conducted to determine the strength of the associations between these variables and abstinence. The results are presented as odds ratio (OR) with 95% confidence interval (CI). A *p* value of < 0.05 was considered statistically significant. Analyses were conducted using IBM SPSS Statistics 28 [45].

## Results

Among the 283 patients who entered OMT treatment at T0, 179 were included at follow-up. Of these 179 patients, 131 were not in a controlled environment for the 30 days prior to T1 and were thus included in the outcome analysis. An examination of attrition revealed no significant differences between the included group and the non-included group regarding baseline characteristics (see Supplementary file 1). The majority of patients reported stable living conditions (88%). We note that among the 131 patients included in this analysis, 41% (*N* = 54) had previously been enrolled in OMT, and were thus restarting treatment. The patients’ mean SDS score was > 10, indicating a high severity of substance use at T0. The mean mental distress score was above 1.0, indicating mental distress of clinical concern [38, 40].

When asked about substance use 6 months prior to the baseline interview (T0), the majority of patients (72%) reported illicit opioid use. Those who did not report illicit opioid use either had a history of opioid use disorder (based on previous enrollment or current status as eligible for OMT), or used prescription opioids at treatment entry. Polysubstance use was reported by 75% of patients. Patients reported using an average of three substances in the 6 months prior to entering treatment (M = 3.4 ± 2.6, Mdn = 3). Nearly half of patients reported cannabis use. The use of benzodiazepines without prescription was reported by 42% of patients. Almost one-third of patients reported having used prescribed benzodiazepines. Around one-third of patients reported stimulant use prior to T0. Around two-thirds of patients reported intravenous use in the 6 months prior to starting treatment (Table 1).

### Changes in substance use from T0 to T1

At follow-up, 116 patients were still in outpatient OMT (88.5%), while 15 patients (11.5%) were no longer receiving OMT. Among these 15 patients, 11 (8.4%) were not receiving any current treatment, while 4 (3.1%) had mostly been in OMT, but reported that they had started a different unspecified treatment. Table 2 presents the changes in the usage of reported substances. We examined change in the reported use of a given substance from T0, and whether use was reported at T1. We found significant reductions in the use of 3 out of 6 substance categories: illicit opioids, benzodiazepines with prescription, and alcohol. Compared to baseline, the use of cannabis, stimulants, and unprescribed benzodiazepines was not significantly changed at T1.

**Table 1:** Baseline characteristics, demographics, substance use, and other relevant variables for patients entering opioid maintenance treatment (OMT) (*N* = 131)

**Sociodemographics**		
Age	M (±)	40 (10)
Male	*n* (%)	97 (74)
Stable living conditions	*n* (%)	115 (88)
**Substance use-related T0 variables**		
Severity of dependence^a^	M (±)	10.1 (3.3)
Intravenous use in past 6 months	*n* (%)	90 (69)
Substance using social network	*n* (%)	48 (37)
Number of substances in past 6 months (*N* = 129)	M (±)	3.4 (2.6)
**Mental health**		
Mental distress^b^	M (±)	1.23 (0.88)
**Goal of treatment**		
Rehabilitation with abstinence	*n* (%)	102 (78.5)
Stabilization and better control of substance use	*n* (%)	28 (21.5)
**Medication at T0**		
Buprenorphine	*n* (%)	34 (25)
Buprenorphine and Naloxone	*n* (%)	71 (54)
Methadone	*n* (%)	26 (21)

^a^ SDS - Severity of Dependence Scale

^b^ HSCL-25 - Hopkins Symptom Checklist 25

Missing data: Rehabilitation with abstinence, *N* = 1; Number of substances in the past 6 months, *N* = 2

**Fig. 1:**
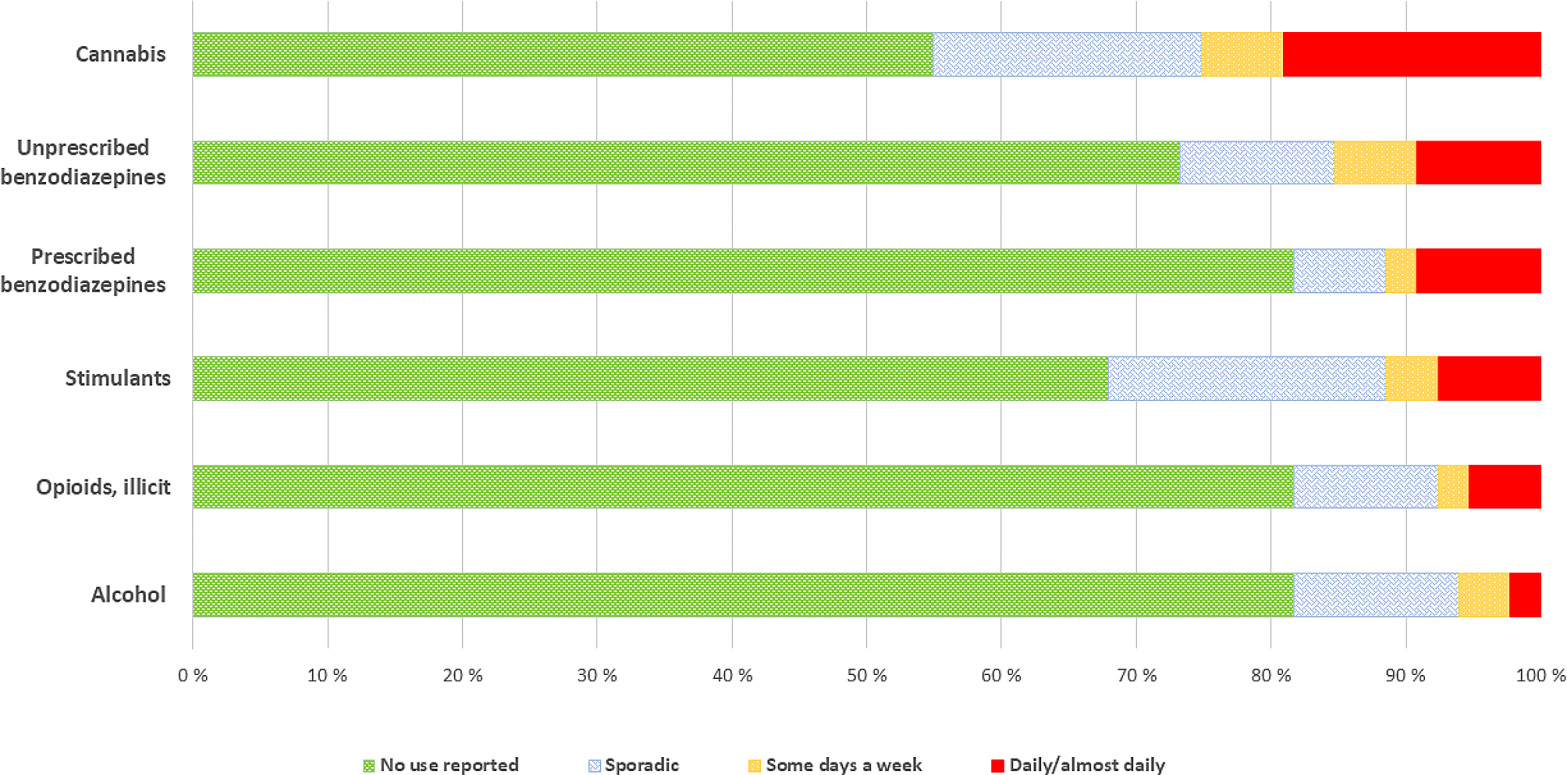
Most used main drug categories within the 4 weeks prior to the 1-year follow-up (T1) (*N* = 131) *Note*: Of *N* = 131 patients, *N* = 116 were still in treatment. “Opioids, illicit” includes heroin and opioids without prescription. “Stimulants” includes amphetamine, cocaine, crack, and other stimulants

Figure 1 presents the frequencies of using the different substance categories at T1. Very few patients reported daily/almost daily non-medical use of opioids. Overall, cannabis was the most commonly daily used substance, followed by benzodiazepines with and without prescription, and stimulants. Alcohol was the least reported daily used substance. About 2 in 5 patients reported intravenous use in the past 6 months at T1.

### Abstinence from concomitant substance use

In the follow-up interview, 31% of patients (*N* = 40) reported drug and alcohol abstinence in the past 4 weeks. Logistic regression was performed to examine factors associated with abstinence (Table 3). Abstinence at T1 was significantly associated with abstinence as a rehabilitation goal expressed at the beginning of treatment (OR = 5.26; 95% CI 1.14–19.55; *p* = 0.013) and increasing age (OR = 1.05; 95% CI 1.00–1.09; *p* = 0.034). The odds of reporting abstinence at follow-up were approximately 5 times higher for patients who stated an abstinence goal at baseline compared to those who aimed to stabilize their substance use.

**Table 2:** Changes in the most used substances past 4 weeks from baseline (T0) to the 1-year follow-up (T1) (*N* = 131)^ab^

	T0		T1			
	Mdn^c^	IQR^d^	Mdn	IQR	z	*p* value^b^
Opioids, unprescribed	5	5	0	0	−6.356	< 0.001
Benzodiazepines, unprescribed	2	4.25	2	2.25	−0.175	0.861
Benzodiazepines, prescribed	5	4	0	2.25	−3.426	< 0.001
Stimulants^e^	1	2	0	2	−1.175	0.240
Cannabis	2	5	1	4	−1.459	0.145
Alcohol	1	5	0	2	−2.154	0.031

^a^ Pairwise comparisons (among patients with descriptions of use on both occasions) of ordinal measurements, ranging from 0 (no use) to 5 (daily use)

^b^*p* values based on Wilcoxon signed rank test

^c^ Mdn = Median

^d^ IQR = Interquartile range

^e^ Stimulants comprise amphetamines, cocaine, crack, methylphenidate, and other stimulants

**Table 3:** Multivariable logistic regression of relevant covariates for association with substance use abstinence at the 1-year follow-up (T1)^a^

Variables	Bivariate analysis OR (95% CI)	p value^b^	Multivariable analysis OR (95% CI)	p value^c^
Age	1.05 (1.01–1.09)	0.016	1.05 (1.00–1.09)	**0.034**
Sex, Male	(ref.)	(ref.)		
- Female	1.34 (0.59–3.08)	0.485		
Severity of dependence^d^	0.93 (0.83–1,04)	0.206		
Mental distress^e^	0.77 (0.50–1.20)	0.250		
Social network: Not substance using	(ref.)	(ref.)		
- Social network: Substance using	0.70 (0.29–1.69)	0.426	0.97 (0.38–2.46)	0.942
- Social network: Mostly alone	2.02 (0.77–5.25)	0.151	2.70 (0.95–7.72)	0.064
Treatment goal: Low-threshold^f^	(ref.)	(ref.)		
- Treatment goal: Rehabilitation w/abstinence	4.55 (1.28–16.10)	0.019	5.26 (1.14–19.55)	**0.013**

^a^ The dependent variable was dichotomized substance use abstinence in the last 4 weeks

^*b*^*p* value obtained from bivariate logistic regression. Results are reported as odds ratios (OR) with 95% confidence intervals (CI).

^c^*p* value obtained from multivariable logistic regression; multivariable analysis included variables with *p* values < 0.20 in bivariate analyses

^d^ SDS - Severity of Dependence Scale

^e^ HSCL-25- Hopkins Symptom Checklist 25

^f^ Missing data: Treatment goals, *N* = 1.

## Discussion

At the start of treatment, the majority of patients reported use of several other substances in addition to opioids. The results at T1 were based on those who were not in a controlled environment. There were no significant differences between the included and non-included groups in terms of baseline characteristics, indicating that the results were relevant for all participants. Our results showed reductions in the use of unprescribed opioids, prescribed benzodiazepines, and alcohol from baseline to the 1-year follow-up. The substance use patterns reported at T1 indicated a small proportion of patients still reporting high frequent (almost daily/daily) concurrent use of other substances while in treatment for OUD. About one-third of patients reported abstinence at T1, i.e., using no substances other than the agonist medication. Abstinence at T1 was significantly associated with age and patients’ treatment goal at baseline.

The concomitant use of substances other than opioids found in this study corroborates the findings of previous research that polysubstance is common in OMT-populations [11, 46, 47]. Among the non-opioid substances, cannabis was the most commonly used substance at baseline, followed by stimulants, illicit benzodiazepines, prescribed benzodiazepines, and alcohol, similar to that reported in previous research in the same context [46]. Roughly 4 out of 10 patients had undergone previous periods of OMT, and had returned to treatment when they were recruited into the present study. At the 1-year follow-up, 11.5% of the included patients had left OMT. This illustrates the complex nature of OUD, and the challenge of facilitating retention in OMT over time.

At follow-up after about 1-year of treatment (T1), the majority of patients reported substantial reductions in illicit opioid use, which is in line with existing research on agonist treatment for OUD [48]. Many patients continued their cannabis and stimulant use throughout treatment, albeit at a lower frequency. At the time the study data were collected, the OMT guidelines advised against benzodiazepine prescription [34]. In the present study frequency of prescribed benzodiazepines use decreased indicating that OMT clinicians followed the guideline-recommended restraint. As such, OMT clinicians may show more restraint regarding benzodiazepine dispensation, compared to GPs or the physicians involved in treatment of OUD patients before OMT initiation. However, we did also observe a pattern of continued use of unprescribed benzodiazepines, similar to findings previously highlighted in the literature [26].

Over the last decade, overall benzodiazepine use has been decreasing in the general population, but signs suggest an increasing trend of benzodiazepine prescriptions in Norwegian OMT [49]. In the revised Norwegian OMT guidelines from 2022, psychosocial treatment and tapering are still strongly recommended for patients with co-occurring benzodiazepine use disorder (BUD) [50]. However, the 2022 guidelines include a new section stating that benzodiazepine substitution/agonist treatment can be considered if certain conditions are met (e.g., long-term BUD, multiple unsuccessful tapering attempts, and high probability of harm reduction). It will be important to examine how these new recommendations affect practice, whether benzodiazepine use in OMT will increase, and how this may affect patients’ treatment outcomes.

In the present patient population, we found that alcohol use appeared to decrease, with the majority of patients reportedly using less alcohol at 12 months after entering treatment. Alcohol use among OMT patients has previously been highlighted as an issue warranting attention [51]. However, reported alcohol use was very low in our study, and the data suggested that it decreased after entering OMT, indicating that this was not a major concern in the present sample.

At follow-up, a sizeable number of patients reported concurrent substance use while in treatment, for example one-third of patients reported stimulants use, and about four out of ten reported cannabis use. This highlights the need for continued focus on other SUDs, in addition to OUD in OMT. However, few patients reported high-frequency polysubstance use (Fig. 1.). For most substances, less than 10% reported daily use at T1, with the exception of cannabis which was used daily/almost daily by about 2 in 10. Rather, most patients reported sporadic or no use of the various substance categories. This analysis reveals a more encouraging picture than if substance use is examined as a dichotomous variable.

Our final regression analysis explored factors associated with abstinence at T1 (apart from OMT medications). Roughly one-third of patients reported abstinence from substance use in the 4 weeks prior to the 1-year follow-up. Our analysis of factors associated with abstinence revealed that an expressed goal of abstinence at baseline was associated with reporting abstinence from non-opioid substances after one year in treatment. This indicates that a patient’s treatment goal has some prognostic value, as has also been found within other SUD categories [52, 53]. As increasing age was associated with abstinence at T1, our findings also suggested that older patients were more likely to use only agonist medication after one year of OMT, i.e., to exhibit abstinence from other substances. We find it encouraging that the risks associated with concomitant use decreased with increasing patient age.

### Clinical implications

Our present results indicate that a treatment goal of rehabilitation with abstinence was associated with abstinence from co-use of other substances after one year in treatment. For patients, treatment goals are not necessarily a static concept, and may change as treatment progress. Treatment providers should be aware of patients’ initial goals, support their efforts to reduce co-use of drugs, and encourage those without such a goal.

### Strengths and limitations

The present study had some limitations. Substance use was self-reported and not independently or biologically verified. Although previous research has shown high agreement between different measures of substance use [54], the possibility of response bias cannot be ruled out. It has been suggested that patients may exaggerate their substance use when there may be some form of reward to gain, especially when seeking treatment [55]. However, in this study, since patients were already in treatment at the time of data collection (T0), the motivation to exaggerate substance use was likely reduced as there were limited benefits associated with doing so. Another potential bias is underreporting substance use while the patient is in a treatment program, as negative consequences may discourage patients from disclosing substance use. Nevertheless, in the present OMT program, patients were not expelled for reporting use of other substances, as stated in the treatment guidelines. Furthermore, the use of external researchers for follow-up interviews, independent of the treatment personnel, likely minimized social desirability bias and improved the validity of the data collected. The form for reporting substance use was limited to the four most used substances. This may have led to underreporting of substance use during the baseline assessment, particularly considering that polydrug use was more frequently reported at baseline. As a result, the actual level of substance use change within the group may have been underestimated. Although the attrition analysis did not reveal any significant distinctions, there may have been differences in other relevant characteristics not measured, such as motivational factors. A strength of this study was that we analyzed data from a clinical cohort, which describes and provides insight into the naturalistic progression of OMT over 12 months [33].

## Conclusions

The majority of patients entering OMT were using other substances in addition to opioids. Outpatient OMT was associated with a reduction of non-medical opioid use. One-third of patients reached perhaps the most optimal goal of OMT within one year of treatment, i.e., abstinence from all substances apart from the opioid agonist medication. The present findings indicated that receiving OMT reduced concomitant substance use. However, a proportion of patients still reported continued and high-frequency substance use at the 1-year follow-up. Clinicians should be aware of these patients’ extended needs, and provide evidence based treatments to motivate these patients to reduce their co-occurring substance use.

### Electronic supplementary material

Below is the link to the electronic supplementary material.

Supplementary Material 1

## Data Availability

The dataset used during the current study is available from the corresponding author on reasonable request.

## Abbreviations

EuropASI: European version of the Addiction Severity Index
HSCL-25: Hopkins Symptom Checklist 25
IQR: Interquartile range
Mdn: Median
NorComt: Norwegian Cohort of Patients in Opioid Maintenance Treatment and Other Drug Treatment
OMT: Opioid maintenance treatment
OUD: Opioid use disorder
±: Standard deviation
SDS: Severity of Dependence Scale
SUD: Substance use disorder
