# Supplementary material for: Change in substance use among patients in opioid maintenance treatment: baseline to 1-year follow-up
Source: Harm Reduct J. 2024 May 24;21:101. doi: 10.1186/s12954-024-01005-x (PMC11127449; doi:10.1186/s12954-024-01005-x)
Supplement: Supplementary file 1 — Supplementary Material 1 [file 12954_2024_1005_MOESM1_ESM.pdf]

Supplementary Table 1. Attrition analysis of baseline characteristics for included and not included patients (N = 283).

	Included (N = 131)	Not included[a] (N = 152)	p-value[b]
**Sociodemographics**			
Age	40 ± 10	39 ± 10	0.165
Male	97 (74)	105 (69)	0.357
Stable living conditions	115 (88)	127 (84)	0.377
**Substance use-related T0 variables**			
Severity of dependence[a]	10.13 ± 3.25	10.64 ± 2.86	0.167
Intravenous use in the past 6 months	90 (69)	118 (78)	0.090
Substance using social network	48 (37)	69 (46)	0.124
Number of substances in past 6 months	3.4 ± 2.6	3.9 ± 3.1	0.265
**Mental health**			
Mental distress[b]	1.23 ± 0.88	1.31 ± 0.84	0.458
**Goal of treatment**			
Rehabilitation with abstinence	102 (78)	110 (73)	0.276
Stabilization and better control of substance use	28 (22)	41 (27)	
**Medication at T0**			
Buprenorphine[c]	33 (25)	37 (24)	0.447
Buprenorphine and Naloxone[d]	71 (54)	82 (54)	
Methadone	27 (21)	30 (20)	
Other	-	3 (2)	

[a] Not included in final analysis due to being lost to follow-up or being in a controlled environment prior to the follow-up interview (T1).
[b] p values from T-tests, or Pearson Chi-Square
Missing data included group: Number of substances in past 6 months: N = 2. Goal of treatment: N =1. Missing data not-included group: Number of substances in past 6 months: N = 3. Goal of treatment: N =1.
